# Underlying Mechanisms and Tunability of the Anomalous Hall Effect in NiCo_2_O_4_ Films with Robust Perpendicular Magnetic Anisotropy

**DOI:** 10.1002/advs.202302956

**Published:** 2023-08-02

**Authors:** Hua Lv, Xiao Chun Huang, Kelvin Hong Liang Zhang, Oliver Bierwagen, Manfred Ramsteiner

**Affiliations:** ^1^ Paul‐Drude‐Institut für Festkörperelektronik Leibniz‐Institut im Forschungsverbund Berlin e. V. Hausvogteiplatz 5–7 10117 Berlin Germany; ^2^ State Key Laboratory of Physical Chemistry of Solid Surfaces College of Chemistry and Chemical Engineering Xiamen University Xiamen 361005 P. R. China

**Keywords:** anomalous Hall effect, ferrimagnetic spintronics, inverse spinel structure, magnetoresistance, perpendicular magnetic anisotropy, skew‐scattering, transition metal oxide

## Abstract

Their high tunability of electronic and magnetic properties makes transition‐metal oxides (TMOs) highly intriguing for fundamental studies and promising for a wide range of applications. TMOs with strong ferrimagnetism provide new platforms for tailoring the anomalous Hall effect (AHE) beyond conventional concepts based on ferromagnets, and particularly TMOs with perpendicular magnetic anisotropy (PMA) are of prime importance for today's spintronics. This study reports on transport phenomena and magnetic characteristics of the ferrimagnetic TMO NiCo_2_O_4_ (NCO) exhibiting PMA. The entire electrical and magnetic properties of NCO films are strongly correlated with their conductivities governed by the cation valence states. The AHE exhibits an unusual sign reversal resulting from a competition between intrinsic and extrinsic mechanisms depending on the conductivity, which can be tuned by the synthesis conditions independent of the film thickness. Importantly, skew‐scattering is identified as an AHE contribution for the first time in the low‐conductivity regime. Application wise, the robust PMA without thickness limitation constitutes a major advantage compared to conventional PMA materials utilized in today's spintronics. The great potential for applications is exemplified by two proposed novel device designs consisting only of NCO films that open a new route for future spintronics, such as ferrimagnetic high‐density memories.

## Graphical abstract

The flexible tunability of anomalous‐Hal‐effect sign and the first discovery of low‐conductivity skew‐scattering make ferrimagnetic NiCo_2_O_4_ films be a new platform for the investigation and manipulation of quantum phenomena in carrier transport. Furthermore, their robust perpendicular magnetic anisotropy even in thick films overcomes a long‐term challenge in today's spintronics, paving a new way for future spintronics, such as high‐density memories.

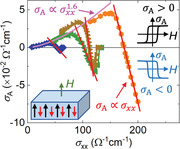

## 1 Introduction

Transition‐metal oxides span a wide range of physical and chemical properties raising fundamental scientific questions and providing variously useful functionalities.^[^ ^1^, ^2^, ^3^ ^]^ As a member of this material class, NiCo_2_O_4_ (NCO) is a promising material candidate for applications in the fields of spintronics, optoelectronics, electrocatalysis, as well as supercapacitors due to its unique properties such as room‐temperature ferrimagnetism, infrared transparency, and rich redox chemistry.^[^ ^4^, ^5^, ^6^, ^7^, ^8^, ^9^ ^]^ For spintronic applications, ferrimagnetic materials are particularly interesting because of their potential to combine the advantages of both ferromagnets and antiferromagnets, the easy control and detection of a net magnetization, as well as antiferromagnetic‐like fast dynamics.^[^ ^10^ ^]^

**Figure 1:**
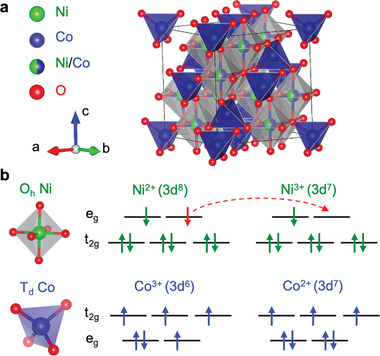
a) Crystal structure of inverse spinel NiCo_2_O_4_, where the tetrahedral cation sites (*T*_*d*_, center of blue sublattice) are occupied by Co, while the octahedral sites (*O*_*h*_, center of gray sublattice) are evenly shared by Ni and Co. b) Possible spin occupations of Ni and Co ions at different valence states, where the dash arrow indicates possible electron hopping between Ni^2 +^ and Ni^3 +^ in the case of their coexistence.

The inverse spinel structure of NCO is based on a crystal lattice that belongs to the space group 227 (**Figure** **1**a) with a face‐centered cubic oxygen sublattice. The tetrahedral (*T*_*d*_) cation sites are occupied by Co while the octahedral (*O*_*h*_) sites are evenly shared by Ni and Co. In the ideal inverse spinel structure, the Ni cations have a Ni^2 +^ valence state, while the Co cations have a Co^3 +^ state assuming the cation configuration ${\left[{\mathrm{Co}}^{3+}\right]}_{T_{d}}{\left[{\mathrm{Ni}}^{2+}{\mathrm{Co}}^{3+}\right]}_{O_{h}}$ (Figure 1b). However, a previous study on our films reveals the occurence of both Ni^2 +^ and Ni^3 +^ valence states on *O*_*h*_ sites, which is accompanied by coexisting Co^3 +^ to Co^2 +^ states on *T*_*d*_ sites leading to the configuration ${\left[{\mathrm{Co}}_{1-x}^{3+}{\mathrm{Co}}_{x}^{2+}\right]}_{T_{d}}{\left[{\mathrm{Ni}}_{1-x}^{2+}{\mathrm{Ni}}_{x}^{3+}{\mathrm{Co}}^{3+}\right]}_{O_{h}}$.^[^ ^11^ ^]^ The relative concentrations of the coexisting valence states in NCO films depend crucially on the synthesis conditions and strongly influence the electrical and magnetic properties of NCO.^[^ ^11^, ^12^, ^13^ ^]^ The resulting high tunability of NCO properties through various strategies is very interesting for the fundamental research and constitutes a clear benefit for the potential spintronic applications. In this respect, it is important to identify the critical material parameters and the corresponding tuning knobs for different synthesis methods.

For studying the magnetic films, the analysis of magnetotransport phenomena, such as the anomalous Hall effect (AHE), is particularly valuable.^[^ ^14^, ^15^ ^]^ However, studies on the AHE in ferrimagnetic materials are rare since previous reports have mainly focused on ferromagnetic materials.^[^ ^16^, ^17^ ^]^ In the framework of such investigations, the occurrence of a very unusual sign reversal in the AHE has been discovered by reducing the thickness of ferrimagnetic NCO films and was therefore ascribed to a finite size effect.^[^ ^14^ ^]^ In general, the AHE originates from quantum coherent band mixing effects beyond the traditional semiclassical transport theory.^[^ ^16^ ^]^ The current knowledge has identified different regimes regarding the scaling relation $\sigma_{A}\propto \sigma_{xx}^{n}$ between the anomalous Hall conductivity (σ_A_) and the longitudinal conductivity (σ_*xx*_). According to this scaling relation, a classification with respect to the underlying AHE mechanism has been established.^[^ ^16^, ^18^ ^]^ However, the comprehensive interpretation of AHE for a specific system is often complicated and there are still many fundamental questions regarding the AHE that remain unclear.^[^ ^16^, ^17^ ^]^ In particular, the understanding of temperature dependence of the AHE is a major long term challenge for experiment and theory.^[^ ^16^, ^17^, ^19^ ^]^ In this respect, ferrimagnetic NCO thin films, with its antiparallel alignment of local magnetic moments and the largely tunable conductivity, could constitute an ideal platform to conduct fundamental studies.

The ferrimagnetic NCO exhibits excellent magnetic characteristics such as strong perpendicular magnetic anisotropy (PMA) even in thick films at room temperature,^[^ ^20^, ^21^ ^]^ which is difficult to achieve by using ferromagnetic materials.^[^ ^10^, ^22^, ^23^, ^24^ ^]^ PMA materials play an important role in today's spintronics, such as magnetic random‐access memories (MRAMs) and perpendicular magnetoresistive sensors.^[^ ^22^, ^25^ ^]^ Compared to MRAMs based on in‐plane anisotropy materials, the utilization of PMA materials leads to much lower power consumption, which is critically important for the commercialization of MRAMs.^[^ ^22^, ^25^ ^]^ Current designs of spintronic devices typically utilize CoFeB (1 ∽ 1.5 nm) thin films and/or multilayers like [Co/Pt]_*n*_ as PMA materials that require a precise control of film thickness in the sub‐nanometer scale.^[^ ^22^, ^23^, ^24^, ^26^ ^]^ Apparently, this requirement constitutes a considerable challenge in the large‐area wafer production leading to high manufacturing costs. Consequently, there is a strong desire for alternative PMA materials, with NCO being a promising candidate. Therefore, the understanding of the PMA in NCO and the evaluation of its potential for device applications are of crucial importance.

In this work, we utilize the magnetotransport measurements to study epitaxial NCO films with different degrees of conductivities, as controlled by the synthesis conditions. The pronounced AHE exhibits an unusual sign reversal that cannot be explained by a finite size effect. Instead, the AHE sign reversal in our NCO films results from a competition between various underlying AHE mechanisms. In this context, an unexpected contribution of skew scattering has been identified in low‐conductivity films, which had previously been observed only in metals with several orders of magnitude higher conductivities. In addition, the ferrimagnetic films exhibit a strong PMA and a high Curie Temperature (*T*_C_), which lies above room temperature for metallic films. Since our work provides insights into magnetic and transport properties of NCO, which are promising for future spintronic applications, we finally propose two novel device concepts with designs consisting only of NCO films.

## 2 Results and Discussion

### 2.1 Longitudinal Transport

The investigated NCO thin films were grown epitaxially on (001)‐oriented MgAl_2_O_4_ (MAO) substrates by pulsed laser deposition (PLD) at different substrate temperatures (*T*_S_) of 325, 350, and 375 °C (See also in Figure S1, Supporting Information). The films with thicknesses (*t*) between 5 and 50 nm exhibit a high structural quality with no impurity phases (Figure S2, Supporting Information) and smooth surfaces with clear atomic terraces (Figure S3, Supporting Information) as revealed by x‐ray diffraction and atomic force microscopy investigations, respectively.^[^ ^11^ ^]^

**Figure 2:**
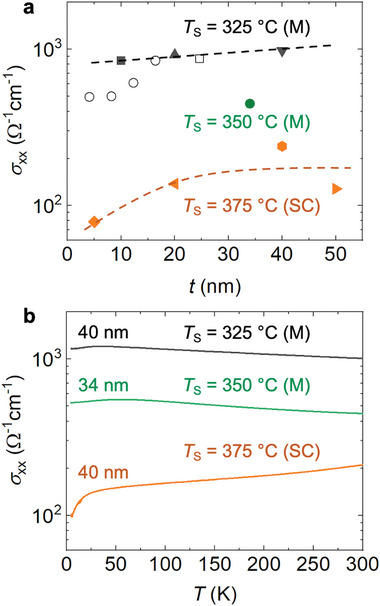
Electrical properties of NCO films. a) Room‐temperature conductivity (σ_*xx*_) as a function of film thickness with different substrate temperatures (*T*_S_), which is comparable with the literature reports (open symbols, *T*_S_ = 320 °C).^[^ ^14^ ^]^ b) Temperature dependent σ_*xx*_ shows a transition between metallic (M) and semiconducting (SC) characteristics tuned by *T*_S_.

**Figure** **2**a illustrates the room‐temperature conductivity of the investigated NCO films. As can be clearly seen, the σ_*xx*_ decreases strongly with increasing growth temperature. In comparison, the dependence on the film thickness is relatively weak, particularly for the samples grown at 325 °C. Figure 2b displays the temperature dependent σ_*xx*_ for samples grown at different substrate temperatures. In accordance with previous studies,^[^ ^11^ ^]^ samples grown at lower temperatures (325 and 350 °C) exhibit metallic (M) transport behavior with a negative temperature coefficient of σ_*xx*_. The detailed temperature dependence can be attributed to electron‐phonon scattering at high temperatures and carrier localization at temperatures below 50 K (See also in Figure S4, Supporting Information).^[^ ^11^ ^]^ Films grown at 375 °C, in contrast, show a semiconducting (SC) characteristic with a positive temperature coefficient of σ_*xx*_. In this case, the transport behavior can be explained by a band conduction model for higher temperatures and a variable‐range hopping model at low temperatures.^[^ ^11^ ^]^ The wide range of conductivities in NCO thin films is governed by the coexistence of Ni^2 +^ and Ni^3 +^ on *O*_*h*_ sites. Thereby, a metallic characteristic is promoted by a large concentration of Ni^3 +^ cations, which leads to the formation of delocalized states at the Fermi level.^[^ ^11^, ^27^, ^28^ ^]^ Indeed, the concentration of Ni^3 +^ cations in our films has previously been found to increase with decreasing growth temperature.^[^ ^11^ ^]^ The feasible tunability of the conductivity over a large range via the growth conditions is beneficial for the design of the NCO‐based applications with various circuit loads, especially important for oxide spintronics.^[^ ^29^, ^30^, ^31^ ^]^

**Figure 3:**
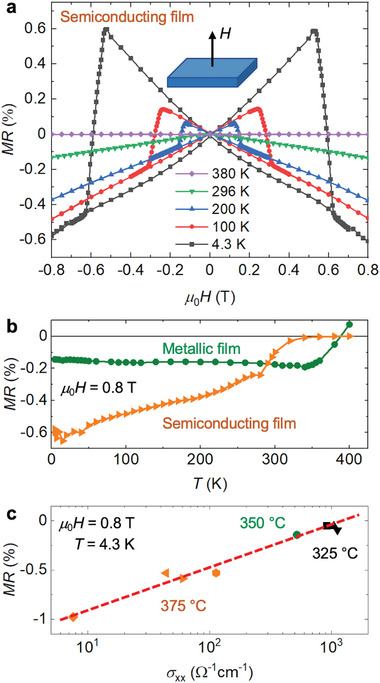
Longitudinal magnetoresistance (*MR*) of NCO films. a) *MR* versus external perpendicular field (*H*) measured at various temperatures of a semiconducting sample (*T*_S_ = 375 °C, 50 nm). b) Temperature dependence of *MR* (0.8 T) displays distinct behaviors in metallic (*T*_S_ = 350 °C, 34 nm) and semiconducting (*T*_S_ = 375 °C, 50 nm) films. c) *MR* versus σ_*xx*_ obtained at 4.3 K from various samples shows a strong correlation.

**Figure** **3**a displays the longitudinal magnetoresistance *MR*, defined as *MR* =[ρ_*xx*_(*H*) − ρ_*xx*_(0)]/ρ_*xx*_(0) through resistivity (ρ_*xx*_) variation under an out‐of‐plane external field (*H*), of a semiconducting NCO film (*T*_S_ = 375 °C, 50 nm) measured at various temperatures. The observed negative magnetoresistance originates from the spin‐dependent carrier scattering,^[^ ^32^, ^33^ ^]^ while the superimposed butterfly‐shaped hysteresis is associated with magnetization reversals in the ferrimagnetic film.^[^ ^33^ ^]^ The temperature dependence of *MR*(0.8 T) exhibits very distinct behaviors for metallic and semiconducting films (Figure 3b). Whereas *MR*(0.8 T) of metallic films remains nearly constant below *T*_C_, a gradual decrease of its absolute value toward room temperature can be seen for the semiconducting films. In accordance with the high structural quality found in our samples,^[^ ^11^ ^]^ a significant contribution from carrier scattering at antiphase boundaries (APBs) can be precluded because of the low absolute values (<1%) and non‐exponential temperature dependence of *MR*.^[^ ^12^, ^34^, ^35^, ^36^ ^]^ The clear correlation between *MR* and σ_*xx*_ shown in Figure 3c indicates that both quantities depend in a similar manner on the relative Ni^3 +^ concentration, which is known to have a crucial impact on the NCO band structure and, thus, also on the magnetoresistance.^[^ ^11^, ^37^ ^]^ The *MR* characteristics reported for different types of single phase NCO films is consistent with the behavior revealed by Figure 3c.^[^ ^14^, ^36^ ^]^ In the case of a pronounced phase coexistence, in contrast, *MR*(σ_*xx*_) with a negative slope is expected.^[^ ^12^ ^]^ Therefore, our analysis of the magnetoresistance constitutes a simple method for the evaluation of the phase purity in NCO films.

### 2.2 Sign Reversal of the AHE

**Figure 4:**
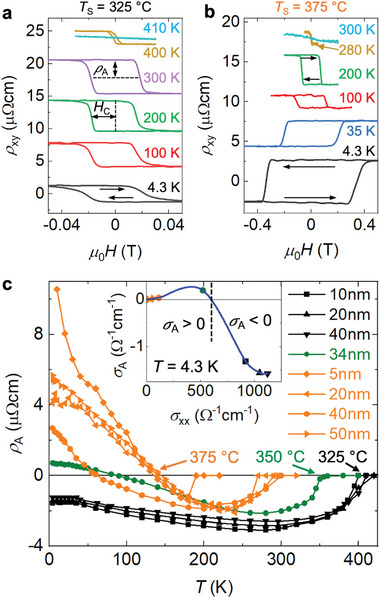
Sign reversal of AHE in NCO films. a–b) AHE signals measured at various temperatures in NCO films (*t* = 40 nm) grown at 325 °C and 375 °C, respectively. c) Temperature dependent ρ_A_ for various NCO films, where the inset displays σ_A_ versus σ_*xx*_ obtained at 4.3 K with $\sigma_{A}=\rho_{A}/\left(\rho_{xx}^{2}+\rho_{A}^{2}\right)$.

One striking characteristics of our NCO films is the occurrence of the AHE with different signs. The NCO films grown at 325 °C exhibit hysteresis loops, which correspond to a negative AHE, defined by a negative (positive) contribution to ρ_*xy*_ upon the positive (negative) magnetization at saturated state, as shown in **Figure** **4**a for a 40‐nm‐thick film grown at *T*_S_ = 325°C. However, for films grown at higher temperatures, the opposite case with hysteresis loops corresponding to a positive AHE is observed at low temperatures, as shown in Figure 4b for a 40‐nm‐thick film grown at *T*_S_ = 375°C. Further results obtained for various samples can be found in Figure S5, Supporting Information. The temperature dependence of the AHE amplitude ρ_A_ is summarized in Figure 4c for various NCO films. For films grown at 325 °C, the ρ_A_ remains negative in the whole temperature range and finally vanishes at the Curie temperature. In contrast, films grown at elevated temperatures (350 and 375 °C) exhibit an AHE sign reversal between 50 and 150 K, a phenomenon that is rarely reported in single phase materials.^[^ ^38^, ^39^, ^40^, ^41^ ^]^ In the case of NCO, a sign reversal has previously been observed only for very thin films with a finite‐size effect being mentioned as its origin.^[^ ^14^, ^42^ ^]^ However, our work reveals the crucial influence of the NCO conductivity σ_*xx*_ on the AHE sign reversal (see inset of Figure 4c) with a negligible influence of the film thickness. In fact, our samples with thicknesses of 5 and 50 nm grown at *T*_S_ = 375°C exhibit sign reversals at the same temperature (≈ 150 K). For the NCO films reported in Ref. [14], a decrease in the film thickness is accompanied by a decrease in the conductivity (open symbols in Figure 2a). The AHE sign reversal in their case actually occurs for films where the conductivity is comparable to our films grown at 350 °C. Consequently, our finding regarding a conductivity‐driven AHE characteristics is consistent with the previously reported observation of a positive (negative) AHE for smaller (larger) thickness exhibiting a lower (higher) conductivity.^[^ ^14^, ^42^ ^]^ In Ref. [42], it is emphasized that the variation of the NCO film thickness can be utilized for the design of application‐specific magnetic properties. Our work clearly demonstrates that the film conductivity is actually the underlying control parameter that can be tuned by the growth conditions independent of the film thickness. Based on the minor thickness dependence, a competition between surface and bulk scattering mechanisms is ruled out as the origin of the AHE sign reversal.^[^ ^40^ ^]^ Furthermore, we also exclude a possible phase transition as the origin of the AHE sign reversal, since ρ_xx_ (Figure 2b), ρ_A_ (Figure 4c), *MR* (Figure 3b) as well as *H*_C_ (Figure S6, Supporting Information) exhibit a smooth temperature dependence in the range where the sign reversal occurs. Consequently, a more detailed study of the underlying AHE mechanisms is needed to reveal the origin of the sign reversal in our NCO films.

### 2.3 AHE Mechanisms in NCO

**Figure 5:**
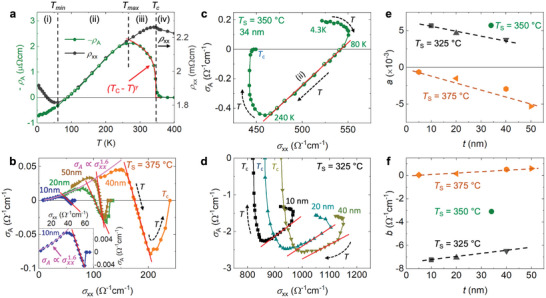
AHE mechanisms in NCO films. a) Comparison of temperature dependent ‐ρ_A_ (blue) and ρ_xx_ (black) of a NCO film (*T*_S_ = 350 °C, 34 nm), where the red line shows the fit curve by assuming ρ_A_∝(*T*_C_ − *T*)^γ^ while the temperature is approaching *T*_C_. b–d) The scaling relation, σ_A_ versus σ_*xx*_, obtained for samples with *T*_S_ = 375 °C, 350 °C, and 325 °C, where the red and pink lines are guides to the eye for σ_A_∝σ_*xx*_ and $\sigma_{A}\propto \sigma_{xx}^{1.6}$, respectively. The inset in b) zooms in the results of 10 nm‐thick sample. e–f) The obtained skew‐scattering coefficient *a* and intrinsic and/or side‐jump contribution *b* from linear fit using Equation (1).

For a better understanding of the general transport characteristics in our NCO films, we compare in **Figure** **5**a the temperature dependence of ‐ρ_A_ (blue) and ρ_*xx*_ (black) for a metallic sample (*T*_S_ = 350 °C). We can clearly identify four different regimes, as indicated by vertical dashed lines:

(i) Low temperature regime at *T* < *T*_min_, where *T*_min_ ≈ 50 K is the temperature at which ρ_*xx*_ reaches its minimum value. In this range, ρ_A_ and ρ_*xx*_ display opposite temperature dependencies. While ρ_A_ changes monotonically, ρ_*xx*_ exhibits an upturn below *T*_min_. The low‐temperature behavior of ρ_*xx*_ is attributed to the quantum correction caused by electron‐electron interaction (EEI) showing a $\sqrt{T}$ dependence of σ_*xx*_ (Figure S4b, Supporting Information) as expected theoretically, whereas the weak localization effect can be excluded here since the observed ρ_*xx*_ upturn is not suppressed by the magnetic field (Figure S4a, Supporting Information).^[^ ^43^, ^44^ ^]^ Generally, the understanding of the EEI influence on electron transport in magnetic systems still poses a great challenge.^[^ ^19^, ^44^, ^45^ ^]^ Thereby, studies of low‐temperature quantum corrections to the AHE mostly focus on weak localization.^[^ ^46^, ^47^, ^48^, ^49^ ^]^ However, quantum correction by EEI to both longitudinal transport and AHE have been reported recently for a spinel ferromagnet.^[^ ^19^ ^]^ Nevertheless, our study provides clear evidence for the absence of EEI corrections to the AHE in the NCO films, which constitutes an interesting fact for future studies on quantum corrections to low‐temperature transport mechanisms.
(ii) Intermediate temperature range at *T*_min_ < *T* < *T*_max_ in which |ρ_A_| reaches its maximum value at *T*_max_. In this range, ρ_A_ and ρ_*xx*_ exhibit a similar temperature dependence, as discussed below in more detail.
(iii) Strong spin‐fluctuation regime *T*_max_ < *T* < *T*_C_ in which ρ_A_ decreases dramatically toward *T*_C_. Despite the strong spin fluctuations near *T*_C_,^[^ ^33^ ^]^ the temperature dependence of ρ_A_ can be well fitted by a power law (*T*_C_ − *T*)^γ^ (red line) with an obtained exponent of γ ≈ 0.2 to 0.4 (see also in Figure S7, Supporting Information), following the behavior of the magnetization commonly observed for ferro‐ and ferrimagnetic materials.^[^ ^50^, ^51^ ^]^ Consequently, the deviation between ρ_A_ and ρ_xx_ in this range is mainly due to the decreasing magnetization, which predominantly affects the AHE.
(iv) Non‐magnetic or paramagnetic regime *T* > *T*_C_ in which the AHE vanishes along with the magnetization whereas ρ_*xx*_ remains finite.^[^ ^52^ ^]^

To gain a comprehensive understanding of the underlying AHE mechanisms in our NCO films, we consider the fact that the AHE may either originate from intrinsic contributions (i.e., Berry curvature) or extrinsic contributions (i.e., side jump and skew scattering), which can be distinguished experimentally by the scaling behavior $\sigma_{A}\propto \sigma_{xx}^{n}$.^[^ ^16^ ^]^ To verify the scaling relation, we mainly consider the intermediate temperature regime ii) in Figure 5a in which a correlation between σ_A_ (ρ_A_) and σ_*xx*_ (ρ_*xx*_) appears to be evident.^[^ ^16^ ^]^ The conductivities of our NCO films fall into the bad‐metal regime (σ_*xx*_ < 10^4^ Ω^−1^cm^−1^) for which a scaling relation of $\sigma_{A}\propto \sigma_{xx}^{1.6}$ would be expected according to previous studies.^[^ ^16^, ^18^ ^]^ As shown in Figure 5b, films grown at 375 °C indeed exhibit a contribution proportional to $\sigma_{xx}^{1.6}$ in the low‐conductivity (low‐temperature) range, which is attributed to the bad‐metal‐hopping mechanism in accordance with the variable‐range hopping‐dominated temperature dependence of resistivity (Figure 2b). In contrast, for the high‐conductivity (high‐temperature) range of the same sample, a linear relationship between σ_A_ and σ_*xx*_ is found (see Figure 5b). In addition, a linear dependence has also been observed for NCO films grown at lower temperatures exhibiting higher conductivities (see Figure 5c,d). The linear scaling relation originates from the fact that both σ_A_ and σ_*xx*_ are proportional to the Bloch state transport lifetime τ, which is further associated with the skew scattering mechanism.^[^ ^16^ ^]^ In fact, such an extrinsic contribution of skew scattering has previously been observed only in super‐clean metals with high conductivity (σ_*xx*_ > 10^6^ Ω^−1^cm^−1^)^[^ ^16^, ^18^ ^]^ and was not found in previous work on NCO.^[^ ^14^, ^42^ ^]^ In our case, we can write:^[^ ^16^ ^]^

(1) $$\sigma_{A}=a\sigma_{xx}+b$$

where the first term describes the contribution from skew scattering with *a* being the skew scattering coefficient. Intrinsic and/or side‐jump contributions are represented by the constant *b*. Figures 5e,f summarizes the contributions of the different AHE mechanisms obtained by linear fitting in the temperature regime ii) according to Equation (1) for various films. As can be clearly seen, the constant *b* exhibits a strong dependence on *T*_S_. For films grown at 325 °C, skew scattering (*a*σ_*xx*_) contributes with a positive sign. At the same time, *b* is negative in sign but larger in absolute value for the whole temperature range which renders a AHE sign reversal impossible [see Equation (1)]. On the other hand, the occurrence of a sign reversal is enabled by the relatively weak intrinsic and/or side‐jump contributions (see contribution *b* in Figure 5f) in films grown at higher temperatures (350 and 375 °C).

Regarding the crucial parameter *b*, it is impossible to discriminate intrinsic and side‐jump contributions by direct current (DC) measurement due to their identical scaling laws.^[^ ^16^ ^]^ Nevertheless, the side‐jump contribution should correlate with the concentration of defects acting as scattering centers, while an opposite behavior is expected for the intrinsic contribution. In our samples, it is revealed that elevated growth temperatures lead to a pronounced increase in the concentration of defects such as oxygen vacancies, which can be expected to contribute to the AHE via side‐jump processes.^[^ ^53^ ^]^ However, the opposite dependence of parameter |*b*| on growth temperature rules out a large proportion of side‐jump events in our case. Consequently, the decrease of |*b*| with increasing growth temperature (see Figure 5f) indicates a dominating intrinsic contribution, consistent with a pronounced variation in the band structure.^[^ ^11^ ^]^ Summing up, the sign change of the AHE is explained as a result of the competition between skew scattering and an intrinsic contribution, where the later becomes particularly dominant for metallic films grown at low temperatures.

The absolute value of the skew scattering constant *a* is in the range of several 10^−3^ (see Figure 5e) that is comparable to values reported for high conductivity samples like Ni, Co, and Fe (‐0.0018,^[^ ^41^ ^]^ 0.0015,^[^ ^54^ ^]^ and ‐0.0037,^[^ ^55^ ^]^ respectively). This coincidence further confirms the occurence of skew scattering in our samples.^[^ ^16^ ^]^ Since NCO is the first material for which skew scattering in the low conductivity regime is revealed, it is important to understand the origin of this transport phenomenon. However, it is difficult to find a detailed explanation for such a quantum phenomenon since it involves, in general, a complex interplay between band‐structure details at the Fermi level, the defect potentials of the involved scattering centers, as well as the type of scattering process. According to Kondo's model, skew scattering may arise from the spin‐dependent scattering of free carriers by localized magnetic moments of defect centers.^[^ ^56^ ^]^ In NCO films, cation antisites induce such localized moments that could act as scattering centers. These antisites are formed by Ni^3 +^ (Co^2 +^) cations on Ni^2 +^ (Co^3 +^) sites of the perfect inverse spinel structure (see Figure 1). In our NCO films, the strong dependence of the conductivity on the growth‐temperature is accompanied by a pronounced variation in the coexistence of different antisite defects.^[^ ^11^ ^]^ Consequently, it is reasonable to identify the cation antisites as the scattering centers responsible for the skew‐scattering contribution to the AHE. Regarding the absolute value and the sign of the skew‐scattering coefficient *a* (see Figure 5f), it might be important to consider that Co cations on *T*_*d*_ sites and Ni cations on *O*_*h*_ sites induce magnetic moments of opposite signs and different absolute values (see Figure 1b). Sign reversals in the skew‐scattering coefficient induced by competing contributions of different elements have also been reported for different ferromagnetic alloys.^[^ ^40^, ^41^ ^]^ Furthermore, it is interesting to note that the sign of the skew‐scattering coefficient is opposite for Ni (negative) and Co (positive) samples.^[^ ^41^, ^54^ ^]^

### 2.4 Magnetic Characteristics

**Figure 6:**
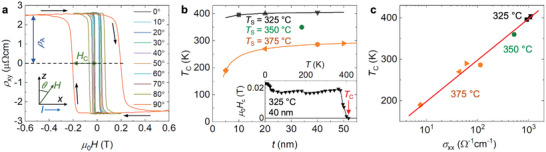
Magnetic properties of NCO films were studied by Hall effect measurements. a) Strong room‐temperature PMA is revealed from AHE characterization with various angle (θ) of applied field (*H*) in a NCO film grown at 325°C (40 nm) as illustrated in the inset, where the arrows indicate the field sweeping directions. b) *T*_C_ as a function of *t* with various *T*_S_, where the lines display the fit curves based on finite size scaling theory, while the inset shows the temperature dependent *H*_C_ used for the *T*_C_ estimation. c) Strong correlation between *T*_C_ and σ_*xx*_ for different NCO samples.

To explore the potential applications of the ferrimagnetic NCO films, we utilize Hall effect measurements, in particular the AHE, to study their magnetic properties. **Figure** **6**a shows the Hall resistivity ρ_*xy*_ measured at room temperature for a sample grown at 325 °C (*t* = 40 nm) with upward and downward sweeps of an external magnetic field. The observed hysteresis loop corresponds to the magnetization switching and its square shape clearly indicates a perpendicular magnetic anisotropy.^[^ ^57^ ^]^ The smooth magnetization switching without any kink strongly indicates the absence of a phase coexistence since additional phases in NCO usually exhibit different coercivities and magnetic anisotropies.^[^ ^35^ ^]^ For a more detailed investigation of the magnetic anisotropy, we measured the dependence of ρ_*xy*_ on the orientation of *H*. Figure 6a displays AHE hysteresis loops for various tilt angles θ, with *H* being parallel to the current flow for θ = 90° as illustrated in the inset. Further results for different in‐plane angle ϕ are shown in Figure S8a,b (Supporting Information). For all field orientations (θ, ϕ), the AHE amplitude ρ_A_ remains the same (Figure 6a; Figure S8c, Supporting Information). Furthermore, the apparent coercive field (*H*_C_) is found to be proportional to (*cos*θ)^−1^ (See Figure S8d, Supporting Information), whereas the shapes of the hysteresis loops are qualitatively identical. This finding provides evidence for a constant out‐of‐plane magnetization and zero in‐plane magnetization at |*H*| > |*H*_C_|, and, thus, a strong uniaxial PMA for the NCO films synthesized under the chosen conditions. In epitaxial NCO thin films, the PMA can be attributed to a tetragonal lattice distortion induced by biaxial strain,^[^ ^27^, ^58^ ^]^ with which has indeed been observed experimentally for our films.^[^ ^11^ ^]^ Our samples exhibit a robust and angle‐independent AHE (see Figure 6a) even at room temperature, while in other PMA materials such a phenomenon has been only reported at low temperatures (2 K).^[^ ^59^ ^]^ Furthermore, the out‐of‐plane magnetization in our NCO films is stable in a much larger range of external in‐plane fields (see Figure S9, Supporting Information) when compared to conventional PMA materials such as CoFeB and multilayers,^[^ ^24^, ^60^, ^61^ ^]^ indicating a much stronger PMA. Since the magnitude of PMA plays an important role in the stability of the memory states,^[^ ^62^ ^]^ the strong PMA at room temperature makes NCO very promising for their potential applications in the MRAMs. At the same time, the coercive field in the commonly used CoFeB thin films is very small, whereas it can be varied in a large range without losing the PMA characteristics by adjusting the growth conditions in our NCO films (see Figure S10, Supporting Information).

Figure 6b displays the *T*_C_ estimated from the temperature dependence of *H*_C_ decay (see inset of Figure 6b; Figure S6, Supporting Information). For samples grown at 325 °C, *T*_C_ ≈ 400 K and *H*_C_ ≈ 0.02 T are comparable to values reported for state‐of‐the‐art single phase stoichiometric films.^[^ ^27^, ^36^, ^63^ ^]^ For films with a strong influence of APBs, for example, a much larger *H*_C_ is expected.^[^ ^36^ ^]^ In this case, the APBs induce the pinning of domain walls (DWs), which hinders their propagation. The interfacial coupling between two adjacent phases via vertical APBs also leads to a higher magnetic field required for the magnetization switching. Furthermore, a significant impact of APBs is not in accordance with the temperature‐independent *H*_C_ observed for our NCO films (inset in Figure 6). A strong influence of APBs, in contrast, leads to an exponential decrease of *H*_C_ with increasing temperature due to thermally activated DW depinning.^[^ ^35^ ^]^ With increasing growth temperature, *T*_C_ strongly decreases leading finally to non‐magnetic films (*T*_S_ > 400°C, not investigated here).^[^ ^11^ ^]^ Accordingly, Figure 6c reveals a clear correlation between *T*_C_ and σ_*xx*_, which can be attributed to the *T*_S_‐dependent valence states. In NCO films with coexisting cation valence states, the electron hopping from Ni^2 +^ to Ni^3 +^ leads to identical initial and final electronic configurations, similar to what has been reported for other materials such as Fe_3_O_4_ and LuFe_2_O_4_.^[^ ^64^, ^65^, ^66^ ^]^ In these cases, the electrons move between the positive ions are itinerant charges, which results in magnetic ordering as well as a finite conductivity.^[^ ^28^ ^]^ In our samples, a low (high) growth temperature results in a relatively large (small) concentration of Ni^3 +^ cations,^[^ ^11^ ^]^ which leads to both a high (low) conductivity^[^ ^28^ ^]^ and an enhanced (reduced) magnetic exchange coupling.^[^ ^13^, ^28^ ^]^ This finding is consistent with the fact that regardless of the chosen variation in the synthesis conditions, higher *T*_C_ values are usually correlated with high conductivities in NCO films.^[^ ^12^, ^13^, ^67^ ^]^ Therefore, the coexistence of cation valence states and the exchange interaction between Ni^3 +^ to Ni^2 +^ are most critical for the transport and magnetic properties.^[^ ^12^ ^]^

### 2.5 Novel Device Designs

In addition to its potential to replace conventional PMA materials in the spintronic applications, NCO also allows for novel device designs enabled by its unique magnetic and transport properties, as revealed by our study. In the following, we propose two applications for which all functional components benefit from the properties of NCO discussed above.

**Figure 7:**
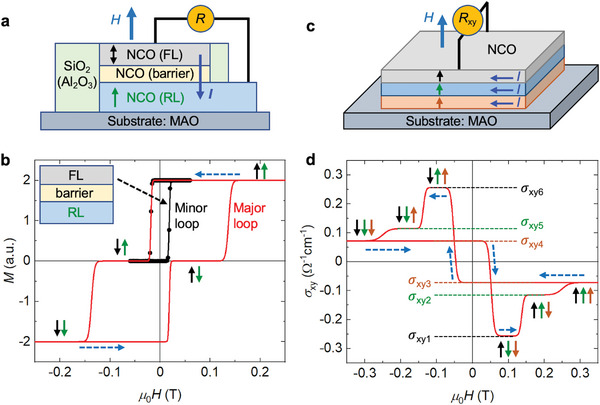
Novel spintronic device designs based on NCO thin films. a) Schematic of a transparent p‐MTJ using NCO layers both as metallic PMA electrodes [free (FL) and reference (RL) layers] and insulating tunnel barrier. b) Simulated room‐temperature magnetic switching characteristic of NCO‐based p‐MTJ, where the dashed arrows indicate the sweeping directions of a perpendicular magnetic field. The magnetization configurations of the FL and RL are indicated by black and green arrows, respectively. c) Schematic of a multiple‐valued logic (MVL) device based on a NCO multilayer structure. For the device functionality, the distinct AHE signals (magnitude and sign) of the individual NCO layers are utilized. d) Red line: Simulated AHE signal, σ_*xy*_ versus *H*, of a trilayer MVL device. Each layer can be switched separately due to large differences in the coercivities, where the six well separated AHE states (σ_*xyi*_ with *i* = 1 to 6, indicated by dashed lines) correspond to the different possible magnetization configurations (perpendicular arrows). Simulation details are described in the Experimental Section.

One promising design is an all‐NCO‐based perpendicular magnetic tunnel junction (p‐MTJ) shown schematically in **Figure** **7**a. The free (FL) and reference (RL) layers consist of metallic NCO films with different coercivities that can be adjusted by the synthesis conditions (see discussion above). The tunnel barrier is formed by nonmagnetic insulating NCO, therefore the entire structure is based on NCO (Figure 7a). Such a MTJ structure with all individual layers consisting of the same material has never been reported or predicted previously. Compared to the previous MTJ designs, for example the one using MgAl_2_O_4_ as the tunnel barrier,^[^ ^68^ ^]^ our proposed all‐NCO‐based structure benefits from an easier and thus more cost‐effective synthesis, which is a crucial aspect for device manufacture and, therefore, potential industrial applications. Furthermore, the perfect lattice matching between the individual layers plays an important role for the electron tunneling^[^ ^69^, ^70^ ^]^ and reduces the risk of dielectric barrier breakdown.^[^ ^71^, ^72^ ^]^ Besides the proper choice of the growth conditions, the insulating characteristics of the NCO barrier can be promoted by the required small thickness.^[^ ^42^ ^]^ The magnetic switching characteristic (see Figure 7b) simulated based on our AHE data is comparable to that of conventional p‐MTJs.^[^ ^73^, ^74^ ^]^ Therefore, such p‐MTJs could be integrated into low‐power‐consumption MRAMs by replacing the currently utilized ferromagnetic p‐MTJ based on CoFeB/MgO/CoFeB. Indeed, exploring novel MTJs is crucial for today's spintronics. Many efforts have been reported recently, such as all‐antiferromagnetic MTJs based on Mn_3_Pt/MgO/Mn_3_Pt^[^ ^75^ ^]^ and Mn_3_Sn/MgO/Mn_3_Sn.^[^ ^76^ ^]^ However, the ferrimagnetic p‐MTJ completely based on NCO shows many advantages compared to these designs: i) The magnetic behavior is much more robust against thickness variations (tolerances in the range of several nm) compared to conventional designs (tolerances of about ±0.3 nm),^[^ ^22^, ^23^ ^]^ which is of crucial importance for large area wafer‐scale production. ii) The efficiency of the spin‐dependent tunneling process benefits from the high spin polarization at the Fermi energy in half‐metallic NCO.^[^ ^11^, ^68^ ^]^ iii) The all‐NCO‐based device is compatible with the concept of transparent and opto‐spintronics (see Figure S1, Supporting Information for information on the transparency of NCO films).

Another application is a multiple‐valued logic (MVL) device, which comprises various NCO films synthesized under different conditions (see Figure 7c). In this case, we utilize the AHE for the functionality of the device and exploit the fact that NCO films with negative and positive AHE can be synthesized. Here, it has to be mentioned that further studies on the synthesis conditions are needed to enable the realization of NCO films with a positive AHE above room temperature. For the current device concept, operation at temperatures below 100 K is assumed (see Figure 4c). Based on our AHE results discussed above, the proposed device structure exhibits multiple well‐distinguished σ_*xy*_ states, as demonstrated by the simulated combined AHE signal shown in Figure 7d. The magnetization of each layer can be switched individually due to the large differences in the respective *H*_C_ value, making the reliable setting of each AHE state feasible. In principle, the number of addressable states is given by 2*N* where *N* is number of NCO layers (2*N* = 6 in our example shown in 7c). This concept might be integrated in high‐density multi‐bit memories that store more than two bits in each cell in a 3D architecture. Current memories, in contrast, comprise only two states per cell, which limits the further increase since it is not possible to keep on shrinking the cell sizes.^[^ ^25^, ^77^ ^]^ Recently, many efforts have been made to develop multiple‐valued memories considering various strategies.^[^ ^78^, ^79^, ^80^, ^81^, ^82^, ^83^, ^84^ ^]^ However, a successful design has not been achieved. In this context, our MVL device concept based on NCO films appears particularly promising.

Finally, to fabricate the all‐NCO‐based devices, it should be considered that the individual layer properties in multilayer structures can be different compared to those of the respective single layers due to gradual changes occurring during the growth of the whole multilayer stack at elevated temperatures. To mitigate the corresponding disadvantages, the oxygen partial pressure could also be utilized for the adjustment of the individual layer properties.^[^ ^11^ ^]^ Since the oxygen pressure can be varied on much shorter time scales compared to growth temperature, the total growth duration could be reduced considerably and, consequently, also the impact of thermally activated processes on the individual layer properties. For the MLV devices (Figure 7c), an incremental decrease of growth temperature from the lowest to the topmost NCO layer should be used whenever possible. Despite the above‐mentioned approaches, more materials engineering is certainly needed to synthesize all‐NCO‐based devices.

## 3 Conclusion

We have investigated the underlying mechanisms and tunability of the magnetic and transport properties of the NCO films grown by PLD. The sample conductivity can be tuned in a wide range via the concentration of Ni^3 +^ valence state by adjusting the synthesis conditions, accompanied by a variation in the entire transport and magnetic characteristics. The sign reversal in the AHE originates from a competition of different underlying transport mechanisms rather than from a finite size effect reported elsewhere. As one of these mechanisms, skew scattering is demonstrated as a remarkable contribution to the AHE, which previously had been reported only for super‐clean metals. With these results, NCO films constitute a new platform for fundamental research of AHE phenomena with tunable AHE signs and low‐conductivity skew‐scattering. In addition, the robust perpendicular magnetic anisotropy and the high Curie temperature, together with the tunability of the film properties via the adjustable conductivity, make NCO a very promising material for spintronic applications. Our proposed NCO‐based device concepts pave new ways for next generation ferrimagnetic and transparent spintronics as well as high density memories.

## 4 Experimental Section

### Sample Growth

Epitaxial NiCo_2_O_4_ thin films were grown using a pulsed laser deposition (PLD) system on double‐sided polished (001)‐oriented MgAl_2_O_4_ substrates. Details of the growth procedure could be found in Ref. [11]. The investigated films were deposited at different substrate temperatures of 325, 350, and 375 °C, respectively. A constant oxygen partial pressure of 50 mTorr was used for all samples during deposition.^[^ ^11^ ^]^ The film thickness varies between 5 and 50 nm by adjusting the deposition time.

### Magnetotransport

Magnetotransport measurements were carried out in the temperature range between 4.3 and 400 K with external magnetic fields up to 0.8 T in vacuum conditions (10^−6^ to 10^−7^ mbar), using a large‐area (5 × 5 mm^2^) van der Pauw (vdP) geometry. For all measurements, a constant direct current (DC) was applied using a Hewlett‐Packard 3245A current source, while the voltage was measured using a Hewlett‐Packard 3458A digital multimeter. During the angle dependence measurement, both field direction and electrical contacts were fixed, where the sample was rotated as shown in the inset in Figure 6a.

### NCO‐Based p‐MTJ Simulation

The simulation of the magnetic switching in all‐NCO‐based p‐MTJs was performed by considering a trilayer structure of NCO(RL)/NCO(barrier)/NCO(FL), as shown in Figure 7a. The insulating NCO barrier NCO was non‐magnetic by choosing a growth temperature higher than 400 °C.^[^ ^11^ ^]^ The FL and RL were supposed to be grown at lower temperatures of 325 and 350 °C, respectively. It assumed that the magnetization hysteresis loops resemble those of the AHE, *M*(*H*)∝ρ_xy_(*H*). Therefore, the total magnetization of the trilayer stack (*M*) was calculated as: *M* = *M*_FL_ + *M*_RL_ by considering the AHE data obtained at room temperature for each layer.

### Multi‐Valued AHE Simulation

The multi‐valued AHE signal was simulated by considering a trilayer structure: NCO(325 °C, 10 nm)/NCO(350 °C, 34 nm)/NCO(375 °C, 40 nm). Hereby, the anomalous Hall conductivity (σ_*xy*_) for the entire stack was assumed as: $\sigma_{xy}=\sum \sigma_{xy}^{j}t_{j}/\sum t_{j}$, where $\sigma_{xy}^{j}$ and *t*_*j*_ were the Hall conductivity and the thickness for layer with the index *j*. The calculation was performed based on the AHE data obtained for each film at 20 K, at which the AHE signals exhibit different signs: negative for NCO(325 °C, 10 nm) and positive for NCO(350 °C, 34 nm) and NCO(375 °C, 40 nm). Various resistance states could be obtained for different magnetization configurations, which could be manipulated individually, while the σ_*xy*_ separation between each state could be freely adjusted by the layer thickness.

## Conflict of Interest

The authors declare no conflict of interest.

## Supporting information

Supporting Information

## Data Availability

The data that support the findings of this study are available from the corresponding author upon reasonable request.
