# Supplementary material for: Underlying Mechanisms and Tunability of the Anomalous Hall Effect in NiCo2O4 Films with Robust Perpendicular Magnetic Anisotropy
Source: Adv Sci (Weinh). 2023 Aug 2;10(28):2302956. doi: 10.1002/advs.202302956 (PMC10558668; doi:10.1002/advs.202302956)
Supplement: Supplementary file 1 — Supporting Information [file ADVS-10-2302956-s001.pdf]

ADVANCED SCIENCE
Open Access

Supporting Information

for *Adv. Sci.*, DOI 10.1002/advs.202302956

Underlying Mechanisms and Tunability of the Anomalous Hall Effect in $NiCo_2O_4$ Films with Robust Perpendicular Magnetic Anisotropy

*Hua Lv, Xiao Chun Huang, Kelvin Hong Liang Zhang, Oliver Bierwagen and Manfred Ramsteiner**

Supporting Information

Underlying Mechanisms and Tunability of the Anomalous Hall Effect in $NiCo_2O_4$ Films with Robust Perpendicular Magnetic Anisotropy

*Hua Lv, Xiao Chun Huang, Kelvin H. L. Zhang, Oliver Bierwagen, and Manfred Ramsteiner**

Dr. H. Lv, Dr. O. Bierwagen, Dr. M. Ramsteiner

Paul-Drude-Institut für Festkörperelektronik, Leibniz-Institut im Forschungsverbund Berlin e. V., Hausvogteiplatz 5-7, 10117 Berlin, Germany

Email Address: ramsteiner@pdi-berlin.de

M.S. X. C. Huang, Prof. Dr. K. H. L. Zhang

State Key Laboratory of Physical Chemistry of Solid Surfaces, College of Chemistry and Chemical Engineering, Xiamen University, Xiamen 361005, People's Republic of China

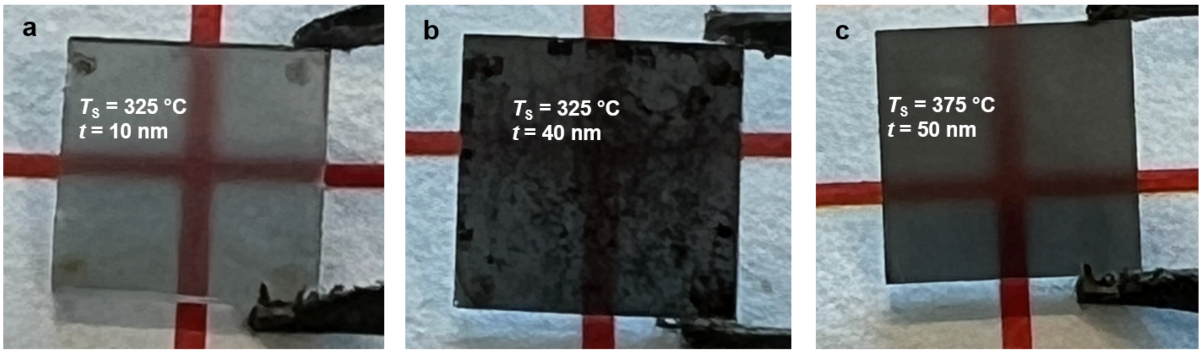

Figure S1. Photographs of $NiCo_2O_4$ (NCO) films grown at different substrate temperatures (T_S) and thicknesses (t): a) T_S = 325 °C, t = 10 nm; b) T_S = 325 °C, t = 40 nm; c) T_S = 375 °C, t = 50 nm. The samples sizes are 5 × 5 mm^2.

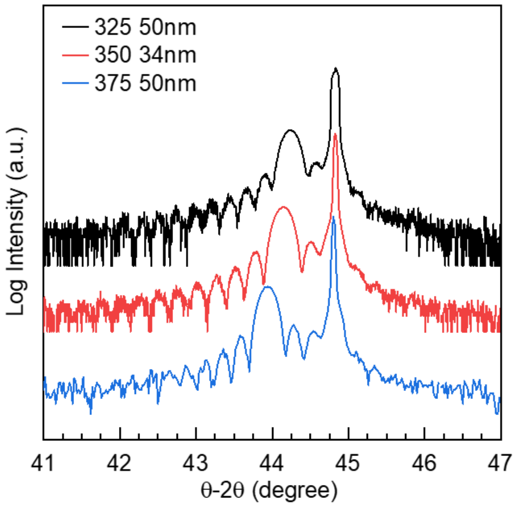

Figure S2. X-ray diffraction (XRD) θ-2θ scans around the (004) reflections of the NCO films grown at different temperatures and thicknesses. The well-defined Kiessig fringes confirm the high crystalline quality and planar character of the surfaces and interfaces of the epitaxial films.

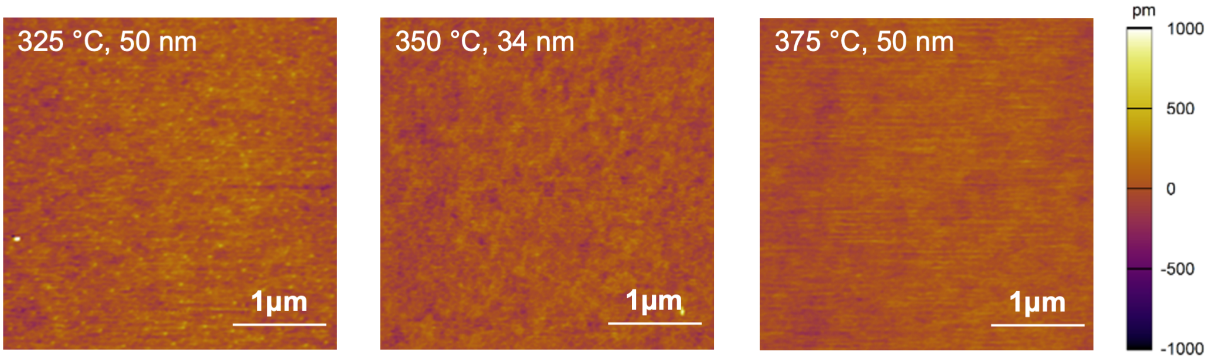

Figure S3. Atomic force microscopy (AFM) image of the NCO films grown at different conditions. The observed atomic steps and terraces confirm the atomically uniform films over a large lateral scale.

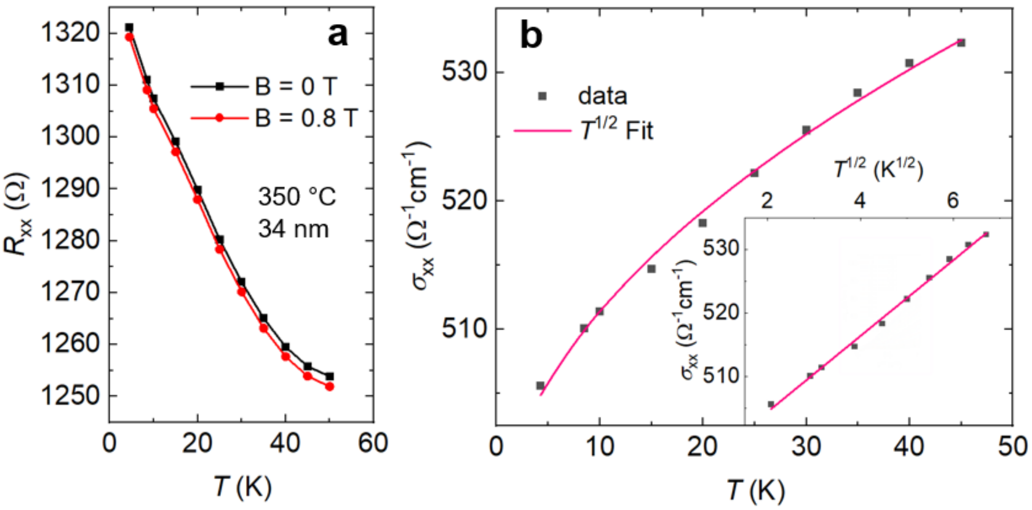

Figure S4. The carrier localization at low temperatures in a $NiCo_2O_4$ thin film (T_S =350 °C, t = 34 nm). (a) Longitudinal resistance (R_{xx}) up-turn with decreasing temperatures occurs at both 0 and 0.8 T, indicating an electron-electron interaction (EEI) origin. The weak-localization can be excluded here since the R_{xx} up-turn is not suppressed by the applied field. (b) The $\sqrt{T}$-dependence of σ_{xx} is consistent with the EEI theory, further confirming the EEI dominated transport behavior in this temperature region.

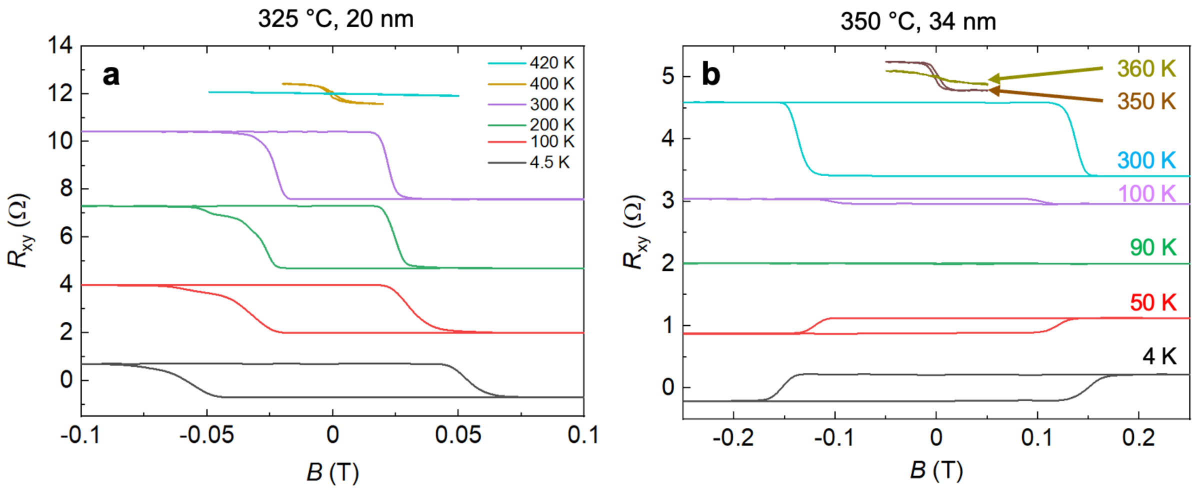

Figure S5. AHE curves measured at various temperatures for NCO films with growth conditions: a) T_S =325 °C (20 nm) and b) T_S =350 °C (34 nm).

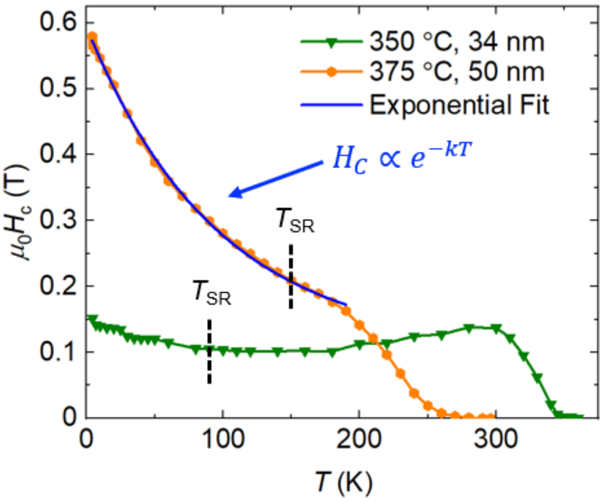

Figure S6. Temperature dependence of H_C for NCO films with T_S =350 °C (34 nm) and T_S =375°C (50 nm), where the blue line shows an exponential fit. The smooth $H_C(T)$ curves exclude possible phase transitions at the temperature of AHE sign reversal (T_{SR}).

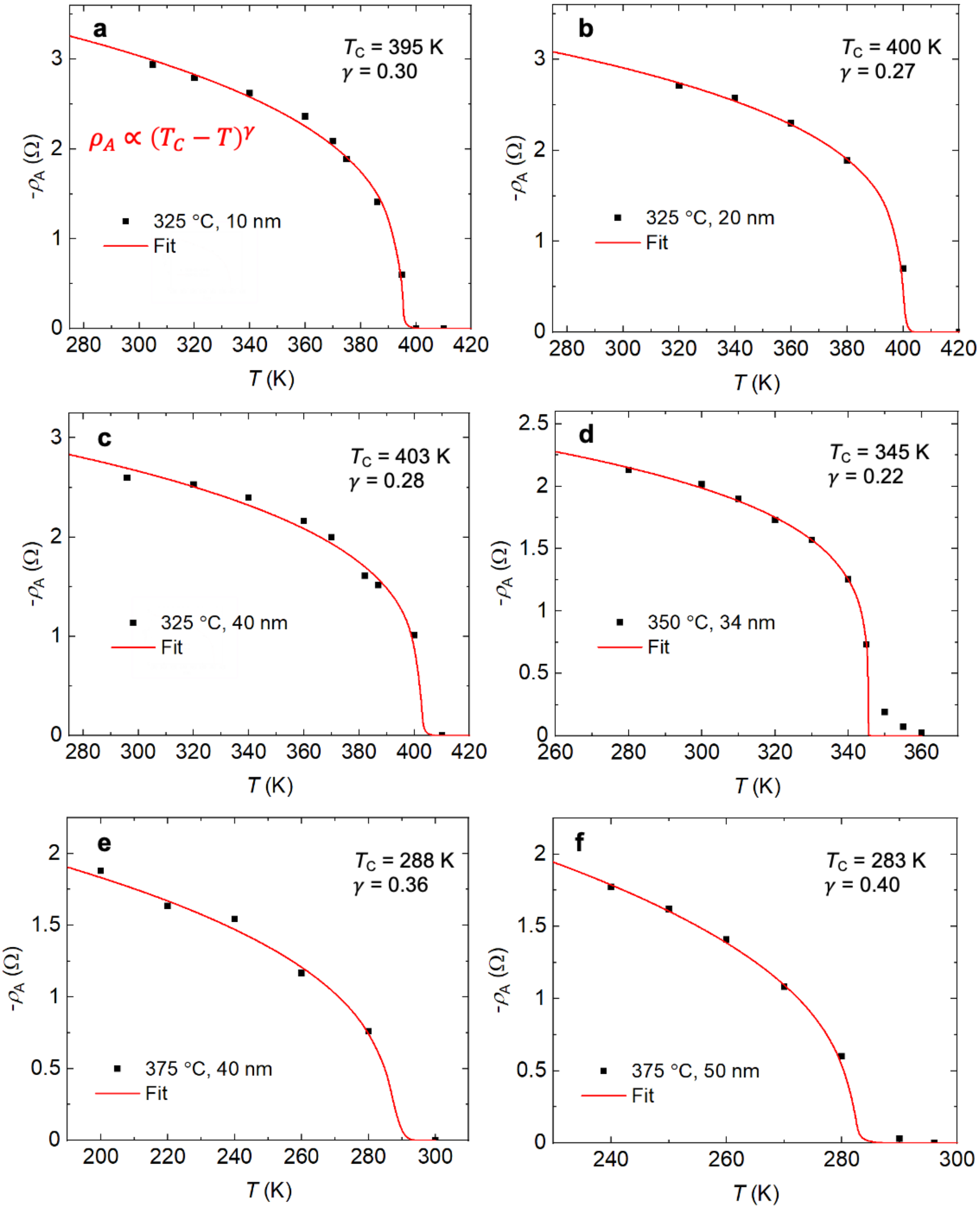

Figure S7. Power law dependences of ρ_A at high temperatures, where the red curves show the fits using $-\rho_A(T) \propto (T_C - T)^\gamma$. The fitting parameter $\gamma \approx 0.2$ to 0.4 is in the range commonly observed for the temperature dependence of magnetization.

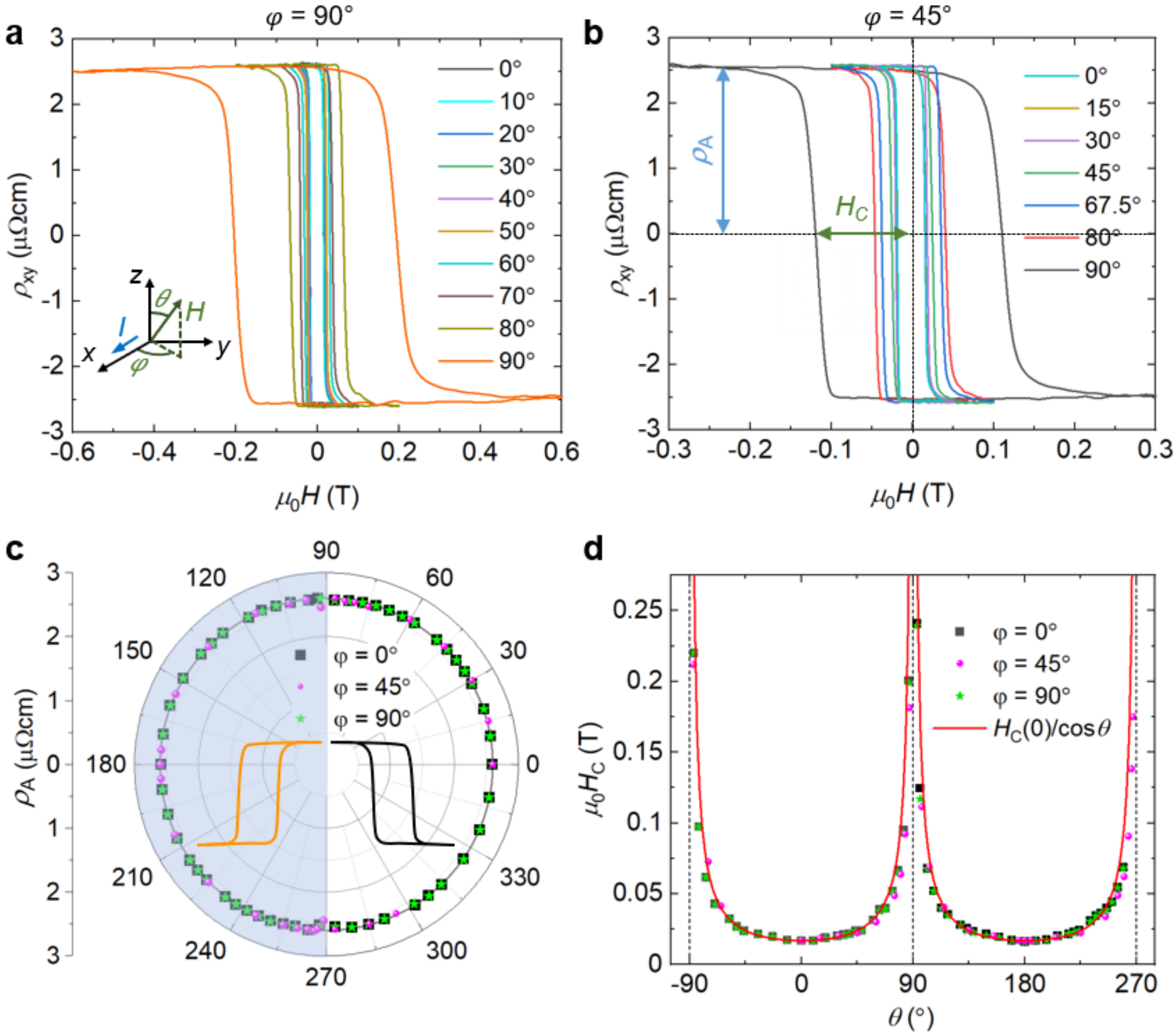

Figure S8. Room temperature AHE measured at various angles of an external field for an NCO film (T_S =325 °C, t = 40 nm). a-b) Tilt angle (θ) dependence of AHE curves measured at different in-plane angles of a) φ = 90° and b) φ = 45°, where the inset in a) shows the measurement configuration. c) The amplitude of the AHE (ρ_A) remains constant in the entire angle range, where the inset indicates the change of AHE curve shapes when crossing the in-plane field condition. d) The coercive field (H_C) dependence on θ at different φ, where the red line is the fit curve using $H_C(\theta) = H_C(0)/\cos\theta$ with $H_C(0)$ as H_C at $\theta = 0°$.

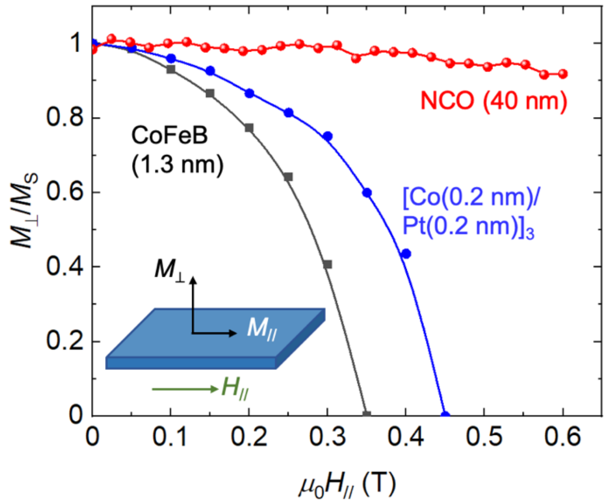

Figure S9. The normalized out-of-plane component of magnetization ($M_{\perp}/M_S$) for CoFeB thin film[1] (black curve) and $[Co/Pt]_n$ multilayers[2] at the room temperature as a function of an in-plane field ($H_{//}$) as illustrated in the inset. For NCO, the normalized AHE resistance ($R_A/R_{A,H=0}$) is shown since this quantity is equal to the normalized out-of-plane magnetization. A slower decay of corresponding $M_{\perp}/M_S$ in the NCO thin film indicates a stronger PMA compared to the conventional ferromagnetic materials, demonstrating its great potential in future spintronic applications.

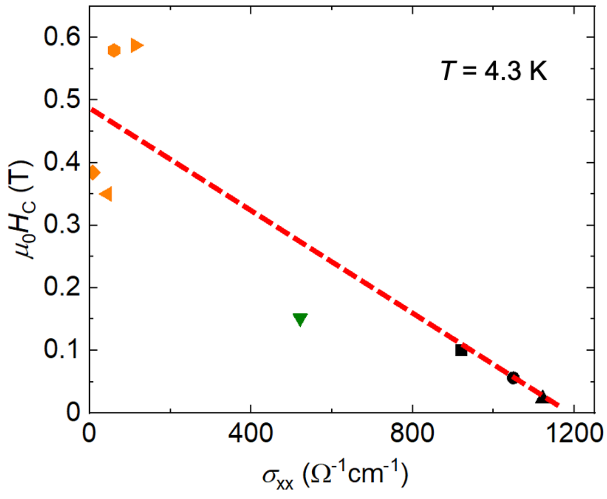

Figure S10. The correlation between H_C and σ_{xx} for various NCO samples (T = 4.3 K), where the red line is a guide to the eye.

Reference:

[1] S. Ikeda, K. Miura, H. Yamamoto, K. Mizunuma, H. D. Gan, M. Endo, S. Kanai, J. Hayakawa, F. Matsukura, H. Ohno, *Nat. Mater.* **2010**, *9*, 721.

[2] T. Das, P. D. Kulkarni, S. C. Puran- dare, H. C. Barshilia, S. Bhattacharyya, P. Chowdhury, *Sci. Rep.* **2014**, 4, 5328.
